# The development and verification of clinical medication pathway for tic disorder in west China

**DOI:** 10.3389/fphar.2025.1682518

**Published:** 2025-11-06

**Authors:** Dan Li, Chunsong Yang, Jianhua Zhang

**Affiliations:** 1 Department of Pediatric Outpatient Nursing, West China Second University Hospital, Sichuan University, Chengdu, China; 2 Key Laboratory of Obstetrics and Gynecologic and Pediatric Diseases and Birth Defects of the Ministry of Education, Sichuan University, Chengdu, Sichuan, China; 3 Key Laboratory of Birth Defects and Related Diseases of Women and Children, Sichuan University, Ministry of Education, Chengdu, China; 4 Department of Pharmacy, Evidence-based Pharmacy Center, West China Second University Hospital, Sichuan University, Chengdu, China

**Keywords:** clinical medication pathway, tic disorder, medication compliance, management strategy, evidence-based medicine

## Abstract

**Background:**

The purpose of this study was to construct a clinical medication pathway for children with tic disorder (TD) and provide a reference for rational drug use for children with TD.

**Methods:**

A literature review was performed to develop an initial clinical medication pathway. Next, a two-round Delphi survey was implemented. A prospective cohort study was carried out to evaluate effectiveness of the constructed clinical medication pathway.

**Results:**

In total, 26 experts (80.8% doctors, 11.5% pharmacists, 7.7% nurses) from Level III medical institutions from west China were included in the Delphi survey. After two rounds of expert consultation, a clinical medication pathway was constructed, comprising 41 items across eight dimensions, these dimensions include participants of the clinical medication management, TD assessment, comorbidity assessment, treatment goals and plans, medication treatment for tics, medication treatment for comorbid attention deficit hyperactivity disorder, recurrence and referral management and medication adherence management. A total of 100 TD patients were consecutively recruited from the outpatient clinic, with 50 cases (8.01 ± 2.49, 35 male) assigned to the clinical medication pathway group and 50 cases (8.25 ± 2.49, 35 male) to the routine treatment group. After 12 weeks of treatment, the clinical medication pathway group showed higher scores in efficacy rate (82% vs. 58%, p = 0.009), YGTSS score (23.86 ± 6.53 vs. 30.68 ± 7.26, p < 0.000), medication adherence (7.47 ± 0.746 vs. 4.32 ± 1.391, p < 0.000) and in the caregivers’ ratings of service quality (4.46 ± 0.706 vs. 4.10 ± 0.839, p = 0.022), service attitude (4.28 ± 0.701 vs. 3.80 ± 0.881, p = 0.003), service efficiency (4.20 ± 0.990 vs. 3.78 ± 1.016, p = 0.039), professional level (4.36 ± 0.631 vs. 3.94 ± 0.956, p = 0.011), and visit satisfaction (4.44 ± 0.675 vs. 3.94 ± 0.956, p = 0.011) compared to the routine treatment group.

**Conclusion:**

This study actively explored clinical medication pathways for children with TD in China and provided a standardized and highly operable medication pathway for reference in clinical practice. This pathway is expected to be widely used in treatment for children with TD. We suggest that further research should update and improve the clinical medication pathway using the latest evidence.

## 1 Introduction

A clinical pathway is a set of standardized treatment models and procedures for a specific disease, including diagnosis, treatment, nursing, rehabilitation, and education. The clinical pathway shows the implementation of the clinical path process in the form of a flowchart that describes the contents of the patient’s admission, examination, treatment, nursing, and health education in detail (Jabbour et al., 2018). The clinical medication pathway is an important part of the clinical pathway and provides systematic and standardized medication options for patients to standardize medication choices, such as timing of medication, dosage, course of treatment, monitoring of adverse reactions, medication management, and timing of withdrawal (Jing et al., 2019). Implementation of a clinical medication pathway can standardize the clinical pathway and ensure safe drug use for patients, reasonably reduce the proportion of medication costs in medical expenses, and improve patient satisfaction. Given the importance of standardized treatment models, it is essential to establish disease-specific clinical medication pathways. Tic disorder (TD) is a condition that could particularly benefit from standardization.

Tics are defined as sudden, rapid, repetitive, and non-rhythmic movements or vocalizations that are often triggered by an urge or premonition in childhood (Grossen, et al., 2024). There are three kinds of TD, transient tic, chronic tic, and Tourette syndrome, with prevalence rates of 1.7%–2.99%, 1.2%–1.61%, and 0.3%–0.77%, respectively (Knight et al., 2012; Yang, et al., 2016; Steinberg et al., 2013). TD is frequently comorbid with other conditions such as attention deficit hyperactivity disorder (ADHD), obsessive–compulsive disorder (OCD), anxiety disorder, depression, and “rage attacks” (sudden, explosive episodes of rage) (Robertson, 2015; Cavanna et al., 2009).

Currently, pharmacological treatment is the most common intervention for patients with TD. To strengthen the management of traditional Chinese medicine (TCM) for TD, the National Administration of Traditional Chinese Medicine formulated the TCM Clinical Pathway for Children with TD in China in 2012 (Wu et al., 2014). Wu et al., evaluated the effect of implementation of this clinical pathway for 132 children with TD in three hospitals, after 24 weeks of intervention, the YGTSS scores were 7.39 ± 2.44 in the clinical pathway group and 19.06 ± 2.87 in the conventional control group (P < 0.05). The TESS scores were 2.05 ± 0.18 and 3.44 ± 0.17 in the clinical pathway group at weeks 12 and 24, respectively, and 9.77 ± 3.82 and 13.50 ± 8.29 in the conventional control group at weeks 12 and 24, respectively (P < 0.05). These statistically significant differences indicate that the clinical pathway group had better effectiveness and safety than the conventional control group (Wu et al., 2014).

Evidence-based medicine provides a scientific framework for developing and optimizing clinical pathways, ensuring that treatment recommendations are supported by the best available evidence. The TCM clinical pathway for TD has been widely used and promoted in China; However, the formulation process and methods of the clinical pathway have not been reported, and recommendations in the clinical pathway lacked support from evidence-based medical knowledge, and the lack of evidence-based methodology in formulating the TCM pathway limits its reproducibility and generalizability. Therefore, developing a standardized, evidence-based clinical medication pathway for TD is essential to ensure rational drug use and improve clinical outcomes. In addition, because of the variability in treatment approaches for TD across different healthcare systems, it is necessary to use evidence-based medicine to systematically formulate a clinical medication pathway to provide a reference for rational drug use for children with TD in China.

## 2 Methods

### 2.1 The construction of clinical medication pathways

#### 2.1.1 Study design

We performed a literature review on MEDLINE, Embase, Cochrane Library, Chinese Biomedical Literature Database, China Knowledge Resource, Integrated Database, VIP Database, and Wanfang Database for literature published from inception to 2024 December For the detailed search strategy, please refer to Supplementary Appendix 1. We included current clinical guidelines addressing TD and focused on pharmacological treatment, overviews of systematic reviews that focused on pharmacological interventions for TD, and medication adherence for TD. The results of these studies were used to design the contents of clinical medication pathway. Then we used the improved Delphi expert consultation method to construct a clinical medication pathway. The clinical medication pathway was registered with the international practice guide registration platform (registration number IPGRP-2020CN146).

#### 2.1.2 Inclusion criteria for experts

The experts included in this study were selected based on the following criteria: (1) Professionals: The experts were clinicians, pharmacists, and nurses specializing in children’s neuropsychiatric diseases. There were no gender restrictions. (2) Institutional Affiliation: The experts were from tertiary hospitals in western China. These institutions are considered high-level medical care providers, offering specialized services for children’s neuropsychiatric conditions. The specific professional fields included children’s neurology, developmental behavior, pediatrics, nursing for children’s neurological diseases, and rational drug use for children’s neurological diseases. (3) Experience: The experts had worked for at least 10 years and had significant experience in clinical diagnosis and treatment of children’s neuropsychiatric diseases. (4) Professional Qualifications: All experts held an intermediate-level professional title or above. (5) Interest: The experts were also selected based on their interest in constructing a clinical medication pathway for children with Tic Disorders (TD).

Recruitment Process: Experts were recruited from a pool of well-established professionals in the field. They were explicitly contacted through a structured invitation, aiming to engage those with significant expertise in the relevant medical and clinical areas. Invitations were sent based on the recognition of their work in the field and their professional standing, ensuring that all selected individuals were acknowledged as well-known experts within their respective specialties.

#### 2.1.3 Content of the expert consultation

Based on the above literature and expert opinion, we designed a consultation questionnaire and organized expert discussion. The preliminary content of the expert consultation for the clinical medication pathway for children with TD included eight categories with 41 items (Supplementary Appendix 2). To ensure the experts fully understood the content of the expert consultation, we introduced the research background, purpose, and related contents before the expert consultation started. We also collected experts’ basic information, including hospital, education background, professional title, clinical working time, hospital grade, and department. The contents of the clinical medication pathway were evaluated using a 5-point Likert scale from 1 (“not important at all”) to 5 (“very important”). We also obtained experts’ opinions about deleting an item and other suggestions. If the average score for items is lower than 4 points or the coefficient of variation was >0.25. The item will be deleted. If experts suggest revisions to items, we will revise the item and conduct a new round of expert consultation.

#### 2.1.4 Statistical methods

##### 2.1.4.1 Experts’ activity

We used the response rate of the consultation questionnaire to evaluate the degree of activity of each expert. The higher the response rate, the greater the importance the experts attached to the consultation. Response rate = number of valid questionnaires/total number of questionnaires * 100%.

##### 2.1.4.2 Experts’ authority

The authority of the experts (q) was evaluated by academic level (q1), judgment reference basis (q2), and familiarity with consulting items (q3). q1 is reflected by the level of professional title, we assigned 1, 0.9, 0.7, 0.5 point to the professional title for Professor, Associate Professor, Intermediate title and others, respectively. q2 is divided into four dimensions: practical experience, theoretical analysis, familiarity with the current situation and intuitive perception. The degree of influence is scored from 0 to 5 points, and the scores are added together as the q2 value of judgment reference basis. q3 is graded from 0 to 5 points and the weight was assigned 1.0, 0.8, 0.6, 0.4. The q-value (authority of the experts) was the average of the above three indicators, namely: q = (q1 + q2 + q3)/3.

##### 2.1.4.3 Expert opinion analysis

Mean ± standard deviation was used as the evaluation index for the degree of concentration of expert opinions. The larger the mean and the smaller the standard deviation, the more concentrated the opinions. The coefficient of variation and Kendall’s W were used to evaluate the degree of coordination of expert opinions. We used SPSS version 22.0 (SPSS Inc., Chicago, IL, USA) for the data analysis. P-values <0.05 indicated statistical significance.

### 2.2 The implementation effectiveness validation of clinical medication pathways

#### 2.2.1 Study design

A prospective cohort study was conducted to evaluate effectiveness of the constructed clinical medication pathway.

#### 2.2.2 Inclusion and exclusion criteria

Inclusion criteria: (1) Patients diagnosed with TD at West China Second Hospital of Sichuan University, with the diagnosis based on DSM-5 criteria; (2) Age under 18 years; (3) Voluntary participation in the study.

Exclusion criteria: (1) Exclusion of other neuropsychiatric disorders such as cerebral palsy, meningitis, speech and motor developmental delay, bruxism, leg rubbing syndrome, myasthenia gravis, strabismus, etc.; (3) Patients or parents unwilling to participate in the study.

#### 2.2.3 Sample size calculation

The sample size for this study was calculated based on the formula for comparing the rate of two independent samples. Parameters were referenced from a clinical pathway study on autism spectrum disorder (Zhao et al., 2020). The clinical pathway group’s overall treatment efficacy was π1 = 0.88, and the control group’s routine treatment efficacy was π2 = 0.54. A significance level of α = 0.05 and a test power of 1 - β = 0.80 were used, with Zα/2 = 1.96 and Zβ = 0.84. The calculation results showed n1 = n2 = 40, so 80 research subjects were required. Considering an expected 20% loss to follow-up, the sample size was expanded to 100 cases.

#### 2.2.4 Intervention and control measures

The intervention group received the TD clinical medication pathway, while the control group followed the routine diagnostic and treatment process.

#### 2.2.5 Collection of baseline data and outcome evaluation indicators

Basic information, including gender, age, course of the disease, type of tics, severity of tics, and complications, was recorded at enrollment. After 12 weeks, follow-up was conducted to assess the effects of drug treatment and patient satisfaction. An improvement rate of >30% on the YGTSS score was defined as effective. Medication adherence was assessed using the Morisky Medication Adherence Scale. Satisfaction was evaluated from five aspects: service quality, service attitude, service efficiency, professional level, and visit satisfaction, with scores ranging from 1 to 5, representing increasing satisfaction.

#### 2.2.6 Statistical methods

Quantitative data were described using mean or median, and comparisons between groups were made using t-tests, analysis of variance (ANOVA), or rank-sum tests. Categorical data were described using proportions, and chi-square (χ^2^) tests were used for binary data. For the efficacy analysis of ordinal data, the Wilcoxon rank-sum test was applied.

#### 2.2.7 Ethical approval

This study was approved by the Ethics Committee of West China Second Hospital of Sichuan University (Ethics number: 201,908). All experts who participated in the construction of the clinical medication pathway for children with TD signed a conflict-of-interest form and declared they had no conflicts of interest. We attended a training and certification session for the Morisky Widget in August 2019 in Beijing, China, and obtained licenses for the use of MMAS-8 from MMAS Research LLC, United States.

## 3 Results

### 3.1 The construction of clinical medication pathways

#### 3.1.1 The result of included studies

We identified 122 citations. After removing duplicates and screening based on titles, abstracts, and full-text articles, we ultimately included 17 studies in this research. For details, see Supplementary Appendix 3. We included five clinical guidelines for TD from China, Japan, Europe, America, Canada (Liu et al., 2020; Yu et al., 2019; Veit et al., 2011; Pringsheim et al., 2019; Pringsheim et al., 2012), three clinical guidelines for TD comorbid ADHD from Canada, America, England (Wolraich et al., 2019; Dalrymple et al., 2020; Stacey et al., 2018), three overviews of systematic reviews (Zhang et al., 2021; Yang et al., 2020; Yang et al., 2016) that focused on pharmacological interventions for children with TD, two studies on medication adherence for children with TD (Yang et al., 2019; Yang et al., 2021), two comparative studies on efficacy in China (Yang et al., 2021; Yang et al., 2020), and two studies about investigation and demand on medication choice for children with TD (Wang et al., 2021; Yang, et al., 2021), and drug data sheets.

#### 3.1.2 Basic information for the experts

We contacted a total of 30 experts from medical institutions in Sichuan province. Four of them declined to participate, and ultimately, 26 experts were included, all of whom worked in Level III hospitals. The basic information of the consulted experts is detailed in Table 1.

**TABLE 1:** Basic characteristics of the included subjects.

Item	%
Gender	
Female	69.2% (18/26)
Male	30.8% (8/26)
Categories of personnel	
Doctors	80.8% (21/26)
Pharmacists	11.5% (3/26)
Nurses	7.7% (2/26)
Academic qualifications	
Bachelor’s degree	57.7% (15/26)
Master’s degree	19.2% (5/26)
Doctoral degree	23.1% (6/26)
job titles	
Professional titles	(53.8%, 14/26)
Associate professional titles	19.2% (5/26)
Intermediate titles	6.9% (7/26)
Years of work experience	4.58 ± 8.95 years

#### 3.1.3 Reliability of expert consultation

All 26 experts completed the first and second rounds of expert consultation, giving a response rate of 100%. All 26 experts had a high degree of authority for the content of this expert consultation, with an average score of 0.89. The coordination coefficients for the two consultation rounds were 0.110 and 0.152 (P < 0.05), indicating that the degree of coordination was relatively acceptable.

#### 3.1.4 First round of expert consultation (Supplementary Appendix 4)

The average score for item 2.6 “Ask patients with TS if they have suicidal thoughts or suicide attempts” was lower than 4 points, and the coefficient of variation was 0.39 > 0.25. This did not meet the set standard. In addition, some experts believe that the incidence of suicide in patients with TS is very low, so they suggest deleting it.

The experts suggested revisions to some items and also added a new item, as follows.

Modified item 1: Experts suggested adding “nurses” to item 1.1 “Establish a ‘doctor-pharmacist-patient-parent-teacher’ treatment alliance to strengthen communication with each other and promote rational drug use,” because nurses are important for health education and medication management for patients with TD.

Modified item 2: Experts suggested that topiramate could also be included in item 5.3 “If the symptom is not well controlled, combined therapy can be used. When the benefit of treatment is greater than the risk, atypical antipsychotics (such as aripiprazole) can be added, starting from the smallest dose.”

Modified item 3: Experts suggested that the treatment period should be longer and revised to 6–12 months in item 5.9 “The treatment cycle for TD is 3–6 months”

Modified item 4: Experts suggested that the monitoring of depressive symptoms should be added for the population using clonidine in the item 8.4 “Focus on monitoring the adverse reactions of the following drugs every month.”

Modified item 5: Experts suggested that the dose should be gradually reduced, and the symptoms should be continuously observed before stopping the drug during the process of drug reduction in item 8.9 “If the symptoms and functions are completely relieved for more than 1 year, the drug can be discontinued carefully after careful evaluation of symptoms, comorbidities and functions, and regular follow-ups should be conducted to monitor condition changes during the period of drug withdrawal.”

New item 1: Experts suggested adding a new item on sleep management and movement management of TD.

#### 3.1.5 Second round of expert consultation (Supplementary Appendix 4)

**FIGURE 1:**
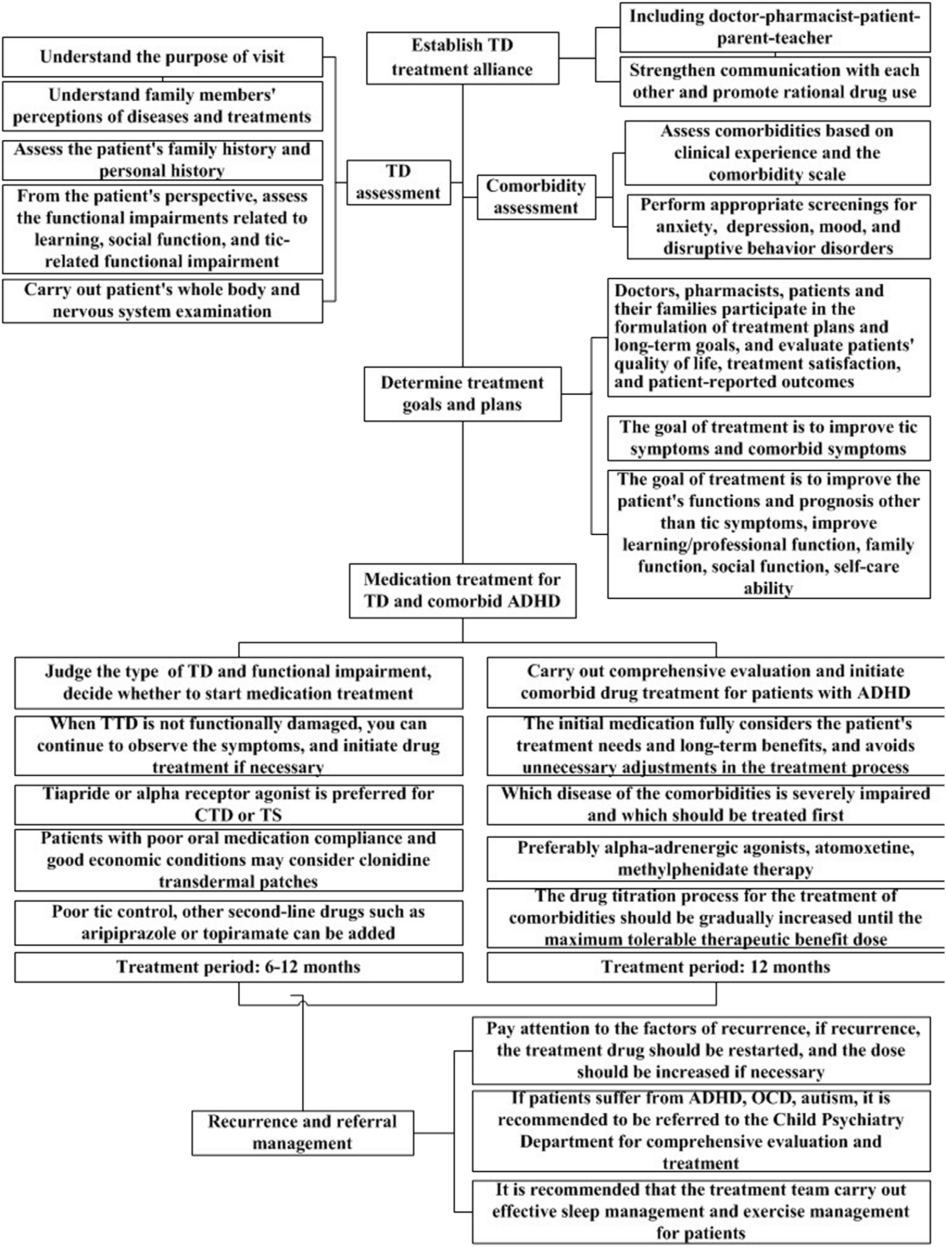
Clinical medication pathway for children with tic disorder.

**FIGURE 2:**
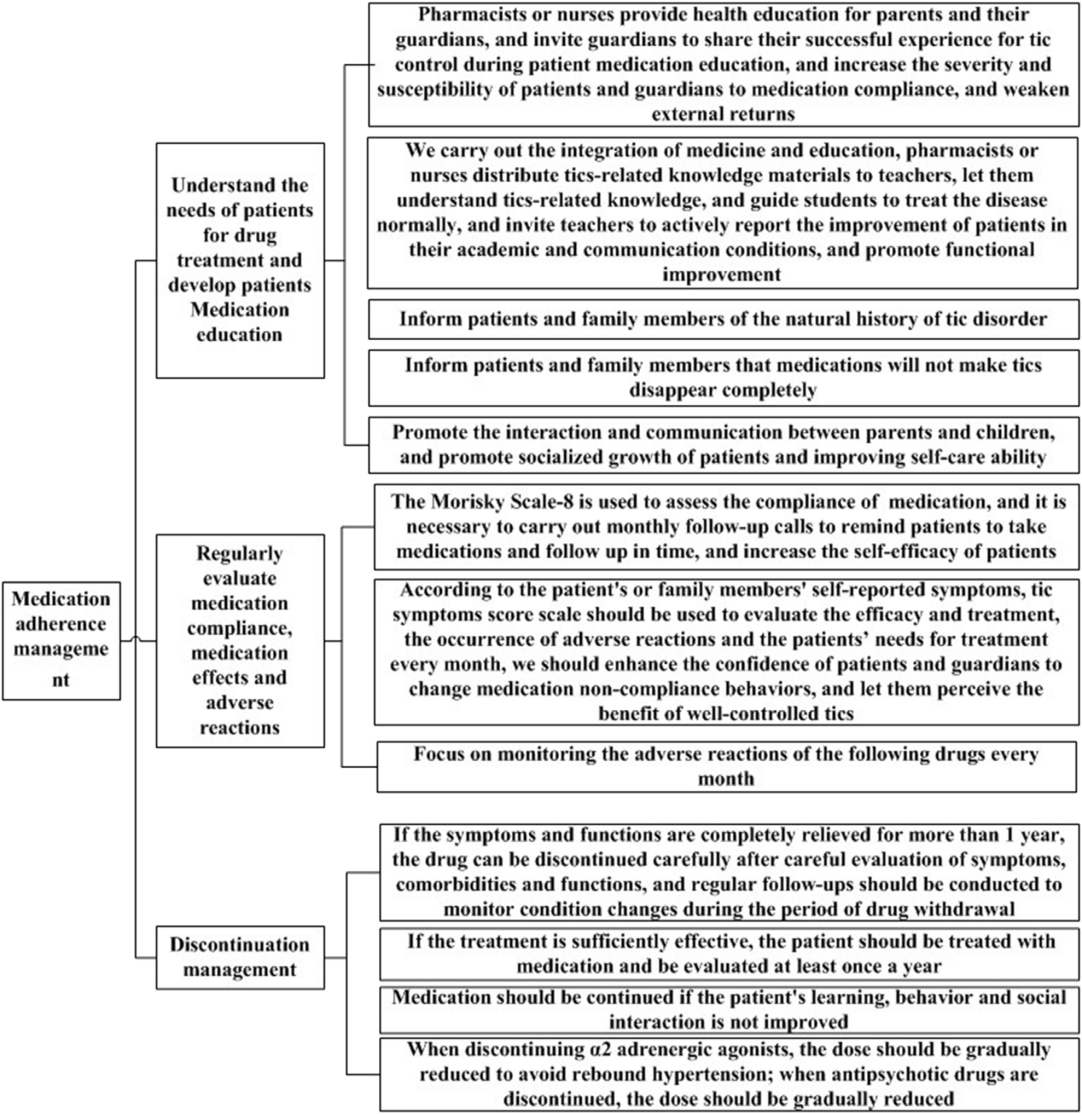
Medication compliance management strategy.

In the second round of expert consultation, there were no items with scores below 4, and all scores for standard deviations and variable coefficients met the requirements. Therefore, no options were deleted, and no items were added or modified. The flow chart for the clinical medication path and medication compliance management strategy for TD developed in this study are shown in Figures 1, 2.

### 3.2 The implementation effectiveness validation of clinical medication pathways

#### 3.2.1 Comparison of baseline characteristics

A total of 100 TD patients were included, with 50 cases in the clinical medication pathway group and 50 cases in the routine treatment group. The two groups were comparable at baseline in terms of age, duration of illness, gender, comorbidities, and baseline YGTSS scores (P > 0.05), as detailed in Table 2.

**TABLE 2:** Description of baseline characteristics.

Items	Clinical medication pathway group (n = 50)	Routine treatment group (n = 50)	F/χ^2^	P
Age	8.01 ± 2.49	8.25 ± 2.49	0.230	0.632
Duration of illness	2.44 ± 1.24	2.53 ± 1.55	0.100	0.752
Gender				
Male	35	35	0.000	1.000
Female	15	15		
Comorbidities ADHD				
Yes	11	11	0.000	1.000
No	39	39		
Family history			0.332	0.564
Yes	6	8		
No	44	42		
Baseline YGTSS score	46.60 ± 10.93	45.50 ± 8.96	0.302	0.584

#### 3.2.2 Efficacy comparison between the two groups

After 12 weeks of treatment, the clinical medication pathway group showed a higher efficacy rate than the control group (P < 0.05), the differences in these indicators (Motor Tics, Phonic Tics, Social Functioning, Total YGTSS) between the two groups were statistically significant, as detailed in Table 3.

**TABLE 3:** Difference in efficacy between the two groups at 12 weeks.

Outcomes	Clinical medication pathway group	Routine treatment group	χ^2^/t	P
Efficacy rates	82%	58%	6.857	0.009
Motor tics	8.38 ± 1.52	9.96 ± 2.17	−4.218	0.000
Phonic tics	8.08 ± 1.16	9.32 ± 1.57	−4.494	0.000
Social functioning	7.40 ± 6.64	11.40 ± 6.70	−2.997	0.003
Total YGTSS	23.86 ± 6.53	30.68 ± 7.26	−4.941	0.000

#### 3.2.3 Medication adherence and satisfaction in the two groups of TD patients

After 12 weeks of treatment, the clinical medication pathway group showed higher scores in medication adherence and in the caregivers’ ratings of service quality, service attitude, service efficiency, professional level, and visit satisfaction compared to the routine treatment group, with P < 0.05, as detailed in Table 4.

**TABLE 4:** Medication adherence and satisfaction between the two groups at 12 weeks.

Evaluation dimensions	Clinical medication pathway group	Routine treatment group	t	P
Medication adherence	7.47 ± 0.746	4.32 ± 1.391	13.828	0.000
Service quality	4.46 ± 0.706	4.10 ± 0.839	2.321	0.022
Service attitude	4.28 ± 0.701	3.80 ± 0.881	3.015	0.003
Service efficiency	4.20 ± 0.990	3.78 ± 1.016	2.094	0.039
Professional level	4.36 ± 0.631	3.94 ± 0.956	2.592	0.011
Visit satisfaction	4.44 ± 0.675	3.94 ± 0.956	2.580	0.011

## 4 Discussion

### 4.1 Findings and clinical value of this study

This study provides a clinically feasible and evidence-based medication pathway that not only addresses the specific medication management needs of TD but also emphasizes medication compliance, which has often been overlooked in previous studies. In the implementation phase, it is essential to adapt the pathway to local healthcare settings, taking into account drug availability, healthcare provider training, and patient education. In clinical practice, behavioral interventions are likely to be beneficial for tics and can be used as a first-line intervention, habit reversal therapy and exposure with response prevention are considered first-line therapeutic options for treating TD, if behavioral therapy does not stop tics, medication can be started (Yang et al., 2016). Many systematic reviews showed that tiapride, clonidine and aripiprazole could be effective and safe for patients with TD (Yang et al., 2020), and these three medications were recommended as the first pharmacological therapy in Chinese guideline (Liu et al., 2020). In addition, a large sample multicenter cross-sectional survey in China presented that the first-line drugs selected by physicians were tiapride (60.74%), clonidine (32.64%), haloperidol (25.62%), aripiprazole (16.53%), and sulpiride (12.4%) for patients with tic disorder and without comorbidities (Yang et al., 2022), so the clinical pathway recommended these medications. The pathway may provide better guidance for medication selection, medication management, and rational medication use for children with TD, and will also standardize the medication behavior of doctors. Clinicians will be able to make the best medication decisions for patients based on the latest progress of evidence-based medicine and patients’ willingness and pay more attention to the entire process of the patient’s medication to provide richer and higher quality drug treatment services. As for other comorbidities, cognitive-behavioral therapy (CBT) with an exposure/response prevention (ERP) component is considered to be the first-line treatment for TD + OCD, If the symptoms are uncontrollable, selective serotonin reuptake inhibitors (SSRIs), such as sertraline, are the first-line pharmacological agents (Liu et al., 2020). For patients with TD who suffer from other significant behavioral disorders, they should be consulted with or referred to professionals in specialized education, psychological intervention, behavioral therapies, and sleep disorders (Liu et al., 2020).

In the analysis of the results of expert consultation, we found that the average score and coefficient of variation of the items were different, which may be related to the experts’ recognition of different items, knowledge level, clinical experience, and the understanding of the current situation of clinical research evidence. But in our study, strict inclusion criteria were set in the process of expert screening, mainly including working years, professional fields and professional titles, to ensure the scientificity and professionalism of consultation. At the same time, in the process of data analysis, the experts’ activity and experts’ authority were also tested statistically. Therefore, the results of expert consultation are also reliable.

### 4.2 Comparison with other studies

In clinical practice, drug therapy is crucial. However, there are no clinical medication pathways for children with tic disorders, so we compared our pathway with those for other pediatric diseases. In 2020, Zhang et al. (Zhang et al., 2020) developed a safe route form for oral administration for children in China, including the following four components. (1) Preparation: medical staff evaluate patient information and prepare for drug delivery. (2) Drug delivery: nurses check patient information, and explain the knowledge related to the use of drugs. After rechecking, medical staff signed their full name and the time on the drug list. (3) Medication: medical staff instruct and assist caregivers in the correct administration of drugs to patients and evaluate the effects of drugs and monitor adverse reactions. (4) Organize the medicine list and medicine cabinet. The management of medication for children with chronic diseases is important. It not only requires the participation of medical staff, but also the participation and cooperation of caregivers. That study showed that the safe route form for oral administration for children could significantly improve the patient’s medication compliance and treatment satisfaction. However, since the path was not targeted at specific disease types, it needs to be further modified and improved to be applied in specific disease fields. Compared with that study, our study targeted children with TD and the clinical medication pathway we developed can therefore provide a reference for improving patient treatment outcomes and satisfaction.

In 2012, Han, (2012) developed a TCM clinical pathway for children with ADHD in China. During the formulation process, a clinical pathway construction team was established to comprehensively search and assess published literature, then the team developed the TCM clinical pathway with reference to the actual situation of medical institutions. In general, the entire formulation process was relatively standardized, but the literature used to support the clinical pathway was not described in detail, and the process and results of the expert consultation were not reported. Compared with that study, our study referred to various types of clinical research evidence to design the specific content of clinical medication pathway for TD and used strict indicators to evaluate the rationality and authority of expert opinions in the process of expert consultation. Therefore, the results of our study are credible.

Rajneesh et al. (2012) developed a clinical treatment pathway for patients with autism and ADHD based on a systematic literature review and expert consensus in the Autism Speaks Autism Treatment Network Psychopharmacology Committee. Their pathway included the systematic evaluation of ADHD symptoms and other comorbidities, behavioral intervention, choice of drug therapy, and precautions. Beth et al. (2012) also established a multidisciplinary team to develop a clinical pathway for insomnia recognition, assessment, and management in children with autism based on systematic evaluation evidence and doctors’ clinical experience. That pathway may help healthcare providers to identify and manage insomnia symptoms in children and adolescents who have autism. The method of constructing the clinical pathway used in the above study was similar to our study, but our study added a medication compliance management strategy, which is essential for the patient’s medication management.

### 4.3 Limitations of this study

This study has the following limitations. (1) The experts participating in the Delphi consultation were all from Sichuan Province, so adaptation may be needed to extrapolate the clinical pathway to other regions. (2) Experts consulted included doctors, nurses, and pharmacists and we did not include other participants, such as patients. (3) This study constructed the clinical pathway based on existing research evidence and expert experience from China, there may be some obstacles in the process of implementing the clinical pathway, such as the accessibility of each drug in different countries, the differences of medication treatment concepts among different doctors, and differences of the price of drugs.

Because we have not verified the implementation effect of this clinical pathway, so it is necessary to conduct clinical study to assess the applicability of the clinical medication pathway in diverse healthcare contexts. Through the implementation of clinical medication pathway, clinical medication can be standardized, treatment effect can be improved, and patient satisfaction can be improved. It is hoped that more doctors can popularize and use this clinical pathway for TD in clinical practice to further test its effect and optimize related content. (4) Due to limitations in funding and time, our study focused primarily on short-term efficacy outcomes. We did not include long-term follow-up, safety indicators, or comprehensive comorbidity assessments in our current study. Future studies could incorporate long-term follow-up and focus on a broader range of indicators. (5) Although we conducted a sample size estimation to meet the minimum statistical requirements, the relatively small sample size of our cohort study is a limitation. This may affect the generalizability of our findings. Future studies with larger sample sizes are needed to further validate our results. Although the clinical medication pathway constructed in this study has guiding significance, there are challenges in promoting it in different clinical environments, such as differences in resources and processes among medical institutions, the need to improve data collection and feedback mechanisms, and the necessity to increase the compliance of patients and their families. We will promote the application of the pathway through multicenter cooperation, professional training, and policy support, and continue to update and improve it.

### 4.4 Conclusion

This study actively explored clinical medication pathways for children with TD in China and provided a standardized and highly operable medication pathway as a clinical practice reference for patients with TD. This pathway is expected to be widely used in the treatment practices for children with TD. It is suggested that further research should update and improve this clinical medication path using the latest evidence-based medicine research evidence. In addition, it is necessary to conduct real world studies to assess the applicability and effectiveness of the clinical medication pathway in diverse healthcare contexts.

## Data Availability

The raw data supporting the conclusion of this article will be made available by the authors, without undue reservation.

## Abbreviations

TD, Tic disorders; ADHD, attention deficit hyperactivity disorder; TCM, traditional Chinese medicine.
