# Supplementary material for: The development and verification of clinical medication pathway for tic disorder in west China
Source: Front Pharmacol. 2025 Nov 6;16:1682518. doi: 10.3389/fphar.2025.1682518 (PMC12631441; doi:10.3389/fphar.2025.1682518)
Supplement: Supplementary file 1 [file Supplementaryfile1.docx]

**Appendix 1 Search Strategy**

**((Pediatric Tourette's Disorder OR Pediatric Tic Disorder OR Pediatric Tourette Syndrome OR Pediatric TD OR Pediatric Motor Tic Disorder OR Pediatric Vocal Tic Disorder OR Pediatric Chronic Tic Disorder) AND (Pharmacological Treatment OR Pharmacological Intervention OR Pharmacotherapy OR Pharmacological Treatment Protocol OR Pharmacological Treatment Strategy) AND (Systematic Review OR Meta-Analysis OR Clinical Guidelines OR Treatment Guidelines OR Expert Consensus OR Practice Guidelines))**

**Appendix2 The preliminary content of expert consultation of clinical medication pathway for children with TD**

| **Framework** | **Specific items** | **Reference basis** |
| --- | --- | --- |
| 1.participants of the clinical medication management (1 item) | 1.1 Establish a "doctor-pharmacist-patient-parent-teacher" treatment alliance to strengthen communication with each other and promote rational drug use | Canadian guideline, Chinese guideline, expert opinion |
| 2.TD assessment (6 items) | 2.1 Understand the patient's purpose of visit (i.e., control symptoms, let the teacher not complain, be able to concentrate in class, make parents happy.) | Expert opinion |
|  | 2.2 Understand family members' perceptions of diseases and treatments | Expert opinion |
|  | 2.3 Assess the patient's family history and personal history | European guideline, Chinese guideline, American guideline, expert opinion |
|  | 2.4 From the patient's perspective, assess the functional impairments related to learning, social function, and tic-related functional impairment | European guideline, Chinese guideline, American guideline, medication adherence study, expert opinion |
|  | 2.5 Carry out patient's whole body and nervous system examination (i.e., electroencephalogram) | European guideline, Chinese guideline, expert opinion |
|  | 2.6 Ask TS patients if they have suicidal thoughts or suicide attempts | American guideline, expert opinion |
| 3.Comorbidity assessment (2 items) | 3.1 Assess comorbidities based on clinical experience and the comorbidity scale (i.e., ADHD, OCD, autism, depression, anxiety) | European guideline, Chinese guideline, American guideline, expert opinion |
|  | 3.2 Perform appropriate screenings for anxiety, mood, and disruptive behavior disorders | European guideline, Chinese guideline, American guideline, expert opinion |
| 4.Treatment goals and plans (3 items) | 4.1 Doctors, pharmacists, patients and their families participate in the formulation of treatment plans and long-term goals, and evaluate patients' quality of life, treatment satisfaction, and patient-reported outcomes | European guideline, Chinese guideline, expert opinion |
|  | 4.2 The goal of treatment is to improve tic symptoms and comorbid symptoms | European guideline, Chinese guideline, expert opinion |
|  | 4.3 The goal of treatment is to improve the patient's functions and prognosis other than tic symptoms, improve learning/professional function, family function, social function, self-care ability | European guideline, Chinese guideline, expert opinion |
| 5.Medication treatment for tics (9 items) | 5.1 If it is a transient tic disorder without functional impairment, it is necessary to continue to observe the patient’s symptoms and initiate drug treatment if necessary | European guideline, Canadian guideline, Chinese guideline, American guideline, expert opinion |
|  | 5.2 In the case of chronic tic disorder and Tourette syndrome, drug therapy can be initiated after weighing the pros and cons, and tiapride or alpha-adrenergic agonists is the first choice | European guideline, Canadian guideline, Chinese guideline, American guideline, expert opinion, comparative study on efficacy, overviews, expert opinion |
|  | 5.3 If the symptom is not well controlled, combined therapy can be used. When the benefit of treatment is greater than the risk, atypical antipsychotics (such as aripiprazole) can be added, starting from the smallest dose | European guideline, Canadian guideline, Chinese guideline, American guideline, expert opinion, overviews |
|  | 5.4 Patients with poor compliance with oral medications and good economic conditions may consider clonidine transdermal patches | Expert opinion, comparative study on efficacy, overviews |
|  | 5.5 Tiapride dose: starting dose 50-100mg/d, therapeutic dose 100-600mg/d | Chinese guideline, expert opinion, comparative study on efficacy, drug instructions |
|  | 5.6 Clonidine dose: starting dose 1mg/week, treatment dose 1-2mg/week | Chinese guideline, expert opinion, comparative study on efficacy, drug instructions |
|  | 5.7 Aripiprazole dose: starting dose 1.25-5.0 mg/d, therapeutic dose 2.5-20.0 mg/d | Canadian guideline, Chinese guideline, expert opinion, drug instructions |
|  | 5.8 The effect of single drug is not good or the tic symptoms are very serious when visiting a doctor, combination drug therapy or second-line drug therapy may be considered | European guideline, Canadian guideline, Chinese guideline, American guideline, expert opinion, overviews |
|  | 5.9 The treatment cycle for TD is 3-6 months | Chinese guideline, expert opinion |
| 6.Medication treatment for comorbid ADHD (6 items) | 6.1 Evaluate the patient's medication history, drug contraindications, height and weight at baseline age, and cardiovascular status before ADHD medication. If medication may affect the QT interval, an electrocardiogram is required | Expert opinion |
|  | 6.2 The initial medication fully considers the patient's treatment needs and long-term benefits, and avoids unnecessary adjustments in the treatment process | European guideline, American guideline, expert opinion |
|  | 6.3 Which disease of the comorbidities is severely impaired and which should be treated first, because the improvement of one symptom will lead to the improvement of another disorder | European guideline, American guideline, expert opinion |
|  | 6.4 When behavioral therapy could not effectively control ADHD comorbidities, and the patient is accompanied by moderate to severe functional impairments, doctors should consider starting drug therapy, preferably alpha-adrenergic agonists, atomoxetine (the initial dose is 0.5 mg/kg/d, then the dose is increased to 1.2 mg/kg/d, the maximum dose is 1.4 mg/kg/d), methylphenidate therapy (methylphenidate sustained release: initial dose 18mg/d, maximum dose 54mg/d), clonidine may bring benefits for two symptoms | Chinese guideline, American guideline, expert opinion, overviews |
|  | 6.5 The drug titration process for the treatment of comorbidities should be gradually increased until the maximum tolerable therapeutic benefit dose | European guideline, Chinese guideline, expert opinion |
|  | 6.6 The treatment cycle for ADHD comorbidities is 1 year | Expert opinion |
| 7.Recurrence and referral management (2 items) | 7.1 Doctors should pay attention to the factors of recurrence, if recurrence, the treatment drug should be restarted, and the dose should be increased if necessary | Chinese guideline, expert opinion |
|  | 7.2 If patients suffer from ADHD, OCD, autism, it is recommended to be referred to the Child Psychiatry Department for comprehensive evaluation and treatment | Chinese guideline, expert opinion |
| 8.Medication adherence management (12 items) | 8.1 Understand the needs of patients for medication, pharmacists or nurses provide health education for parents and their guardians, and distribute tics knowledge booklets to patients and family members to provide with sufficient disease and treatment information; and invite guardians to share their successful experience for tic control during patient medication education, and increase the severity and susceptibility of patients and guardians to medication compliance, and weaken external returns | Canadian guideline, American guideline, expert opinion, medication adherence study |
|  | 8.2 We carry out the integration of medicine and education, pharmacists or nurses distribute tics-related knowledge materials to teachers, let them understand tics-related knowledge, and guide students to treat the disease normally, and invite teachers to actively report the improvement of patients in their academic and communication conditions, and promote functional improvement | Canadian guideline, expert opinion, medication adherence study |
|  | 8.3 Promote the interaction and communication between parents and children, and promote socialized growth of patients and improving self-care ability | Expert opinion, medication adherence study |
|  | 8.4 Focus on monitoring the adverse reactions of the following drugs every month:  (1) Tiapride: dizziness, fatigue, lethargy, gastrointestinal reactions  (2) Clonidine: sedation, orthostatic hypotension, prolonged P-R interval, heart rate monitoring  (3) Aripiprazole: headache, insomnia, irritability, anxiety, drowsiness, gastrointestinal reactions, movement disorders, metabolic disorders and hormone-related adverse reactions | overviews, drug instructions, European guideline, Canadian guideline, Chinese guideline, American guideline, expert opinion, comparative study on efficacy |
|  | 8.5 The Morisky Scale-8 (*) is used to assess the compliance of patients with medication, and it is necessary to carry out monthly follow-up calls to remind patients to take medications and follow up in time, and increase the self-efficacy of patients | Expert opinion, medication adherence study |
|  | 8.6 According to the patient's or family members' self-reported symptoms, tic symptoms score scale should be used to evaluate the efficacy and treatment, the occurrence of adverse reactions and the patients’ needs for treatment every month, we should enhance the confidence of patients and guardians to change medication non-compliance behaviors, and let them perceive the benefit of well-controlled tics | European guideline, Canadian guideline, American guideline, expert opinion, comparative study on efficacy, medication adherence study, investigation |
|  | 8.7 Inform patients and family members of the natural history of tic disorder | Canadian guideline, American guideline, expert opinion |
|  | 8.8 Inform patients and family members that medications will not make tics disappear completely | Chinese guideline, American guideline, expert opinion |
|  | 8.9 If the symptoms and functions are completely relieved for more than 1 year, the drug can be discontinued carefully after careful evaluation of symptoms, comorbidities and functions, and regular follow-ups should be conducted to monitor condition changes during the period of drug withdrawal | Chinese guideline, expert opinion |
|  | 8.10 If the treatment is sufficiently effective, the patient should be treated with medication and be evaluated at least once a year | Chinese guideline, expert opinion |
|  | 8.11 Medication should be continued if the patient's learning, behavior and social interaction is not improved | Expert opinion |
|  | 8.12 When discontinuing α2 adrenergic agonists, the dose should be gradually reduced to avoid rebound hypertension; when antipsychotic drugs are discontinued, the dose should be gradually reduced | American guideline, expert opinion |

Note: TD: Tic disorder; TS: Tourette syndrome; ADHD: attention-deficit hyperactivity disorder; OCD: Obsessive-Compulsive Disorder;

We attended a training and certification session for the Morisky Widget in August 2019 in Beijing, China, and obtained licenses for the use of MMAS-8 from MMAS Research LLC, USA

Records after duplicates removed
(n =40)

Records screened
(n = 82)

Records excluded (n =58)

1. Non-pharmacological intervention (n = 20)
2. Off-topic (n=38)

Full-text articles assessed for eligibility
(n = 24)

Full-text articles excluded, with reasons (n = 7)

(1) literature review (n = 4)

(2)Non-pharmacological intervention studies (n = 3)

Studies included in

this research

(n = 17)

Records identified through database searching
(n = 120)

Additional records identified through other sources
(n = 2)

**Appendix 3: Flowchart of literature search and study inclusion procedure.**

**Appendix 4 Results of the first and second round of expert consultation**

| **item** | **The first round** | | | **item** | **The second round** | | |
| --- | --- | --- | --- | --- | --- | --- | --- |
|  | **Mean** | **standard deviation** | **variable coefficient** |  | **Mean** | **standard deviation** | **variable coefficient** |
| item1.1 | 4.69 | 0.62 | 0.13 | item1.1 | 4.73 | 0.53 | 0.11 |
| item2.1 | 4.73 | 0.53 | 0.11 | item2.1 | 4.77 | 0.43 | 0.09 |
| item2.2 | 4.58 | 0.58 | 0.13 | item2.2 | 4.65 | 0.49 | 0.10 |
| item2.3 | 4.58 | 0.64 | 0.14 | item2.3 | 4.62 | 0.57 | 0.12 |
| item2.4 | 4.65 | 0.63 | 0.14 | item2.4 | 4.69 | 0.55 | 0.12 |
| item2.5 | 4.27 | 0.83 | 0.19 | item2.5 | 4.38 | 0.64 | 0.15 |
| item2.6 | 3.69 | 1.44 | 0.39 |  |  |  |  |
| item3.1 | 4.62 | 0.50 | 0.11 | item3.1 | 4.69 | 0.47 | 0.10 |
| item3.2 | 4.23 | 0.91 | 0.21 | item3.2 | 4.31 | 0.84 | 0.19 |
| item4.1 | 4.69 | 0.55 | 0.12 | item4.1 | 4.69 | 0.55 | 0.12 |
| item4.2 | 4.88 | 0.33 | 0.07 | item4.2 | 4.92 | 0.27 | 0.06 |
| item4.3 | 4.77 | 0.51 | 0.11 | item4.3 | 4.81 | 0.40 | 0.08 |
| item5.1 | 4.54 | 1.03 | 0.23 | item5.1 | 4.65 | 0.69 | 0.15 |
| item5.2 | 4.69 | 0.55 | 0.12 | item5.2 | 4.73 | 0.45 | 0.10 |
| item5.3 | 4.54 | 0.65 | 0.14 | item5.3 | 4.58 | 0.58 | 0.13 |
| item5.4 | 4.73 | 0.53 | 0.11 | item5.4 | 4.77 | 0.51 | 0.11 |
| item5.5 | 4.65 | 0.56 | 0.12 | item5.5 | 4.69 | 0.47 | 0.10 |
| item5.6 | 4.54 | 0.71 | 0.16 | item5.6 | 4.58 | 0.58 | 0.13 |
| item5.7 | 4.46 | 0.76 | 0.17 | item5.7 | 4.50 | 0.65 | 0.14 |
| item5.8 | 4.69 | 0.55 | 0.12 | item5.8 | 4.73 | 0.53 | 0.11 |
| item5.9 | 4.38 | 0.80 | 0.18 | item5.9 | 4.42 | 0.76 | 0.17 |
| item6.1 | 4.69 | 0.47 | 0.10 | item6.1 | 4.73 | 0.45 | 0.10 |
| item6.2 | 4.88 | 0.33 | 0.07 | item6.2 | 4.88 | 0.33 | 0.07 |
| item6.3 | 4.65 | 0.56 | 0.12 | item6.3 | 4.65 | 0.56 | 0.12 |
| item6.4 | 4.65 | 0.63 | 0.14 | item6.4 | 4.69 | 0.55 | 0.12 |
| item6.5 | 4.50 | 0.76 | 0.17 | item6.5 | 4.58 | 0.58 | 0.13 |
| item6.6 | 4.54 | 0.71 | 0.16 | item6.6 | 4.58 | 0.64 | 0.14 |
| item7.1 | 4.58 | 0.58 | 0.13 | item7.1 | 4.58 | 0.58 | 0.13 |
| item7.2 | 4.62 | 0.80 | 0.17 | item7.2 | 4.69 | 0.62 | 0.13 |
|  |  |  |  | item7.3 | 4.73 | 0.45 | 0.10 |
| item8.1 | 4.73 | 0.45 | 0.10 | item8.1 | 4.73 | 0.45 | 0.10 |
| item8.2 | 4.69 | 0.55 | 0.12 | item8.2 | 4.73 | 0.45 | 0.10 |
| item8.3 | 4.69 | 0.55 | 0.12 | item8.3 | 4.73 | 0.45 | 0.10 |
| item8.4 | 4.69 | 0.47 | 0.10 | item8.4 | 4.73 | 0.45 | 0.10 |
| item8.5 | 4.50 | 0.71 | 0.16 | item8.5 | 4.54 | 0.65 | 0.14 |
| item8.6 | 4.65 | 0.56 | 0.12 | item8.6 | 4.65 | 0.56 | 0.12 |
| item8.7 | 4.54 | 0.76 | 0.17 | item8.7 | 4.58 | 0.64 | 0.14 |
| item8.8 | 4.46 | 0.90 | 0.20 | item8.8 | 4.58 | 0.58 | 0.13 |
| item8.9 | 4.62 | 0.64 | 0.14 | item8.9 | 4.65 | 0.56 | 0.12 |
| item8.10 | 4.38 | 0.75 | 0.17 | item8.10 | 4.42 | 0.70 | 0.16 |
| item8.11 | 4.62 | 0.64 | 0.14 | item8.11 | 4.65 | 0.56 | 0.12 |
| item8.12 | 4.73 | 0.53 | 0.11 | item8.12 | 4.77 | 0.43 | 0.09 |
